# Development, evaluation of the PNA RT-LAMP assay for rapid molecular detection of SARS-CoV-2

**DOI:** 10.1038/s41598-021-00041-y

**Published:** 2021-10-14

**Authors:** Chinbayar Bat-Ochir, Yeon-Sook Kim, Han Gyeul Kim, Si Seok Lee, Han Woo Lee, Hee Kyung Park

**Affiliations:** 1 Seasun Biomaterials, Daejeon, Korea; 2 Division of Infectious Diseases, Department of Internal Medicine, Chungnam National University School of Medicine, Daejeon, Korea

**Keywords:** Biological techniques, Molecular biology

## Abstract

Dual-labeled PNA probe used RT-LAMP molecular rapid assay targeting SARS-CoV-2 ORF1ab and N genes was developed, and the analytical, clinical performances for detection of SARS-CoV-2 RNA extracted from clinical nasopharyngeal swab specimens were evaluated in this study. Data showed that this assay is highly specific for SARS-CoV-2, and the absolute detection limit is 1 genomic copy per microliter of viral RNA which can be considered to be comparable to gold-standard molecular diagnostic method real-time reverse transcriptase PCR. Both clinical sensitivity and specificity against a commercial real-time RT-PCR assay were determined as identical. In conclusion, the PNA RT-LAMP assay showed high analytical and clinical accuracy which are identical to real-time RT-PCR which has been routinely used for the detection of SARS-CoV-2.

## Introduction

Severe Acute Respiratory Syndrome Coronavirus 2 (SARS-CoV-2) is a single-stranded RNA virus that causes Coronavirus Disease 2019 (COVID-19) which has been spreading globally at rapid speed and is more contagious than most of other human respiratory tract infectious microorganisms^1, 2^. The high transmissibility demands rapid and accurate detection of SARS-CoV-2 at the early stages of the infection in a cost-effective manner^3^. Highly specific real-time reverse transcriptase PCR assays have been used for the identification of the SARS-CoV-2 RNA globally; however, it requires laboratory-based PCR instruments and needs about 2 h of run-time as well as additional incubation of 15 to 30 min for cDNA synthesis from template RNA^4^.

Loop Mediated Isothermal Amplification (LAMP) is a molecular technique capable of detecting nucleic acids with high sensitivity within a reduced time compared to classical real-time PCR and has been used widely for the detection of viral infections in a time-effective manner^5^. Since beginning of the COVID-19 outbreak, various molecular detection assays based on real-time PCR or LAMP aimed for detection of SARS-CoV-2 nucleic acid have been developed and received Emergency Use Authorizations (EUA) from the US FDA or the authorizations of the regulatory agencies in the country of production. However, quite a few LAMP tests have been commercialized successfully and multiple scientific reports have shown that the LAMP tests have high enough analytical and clinical performances^6–8^, numerous reports have been showing that the LAMP assays still have some challenges; for instance, poor specificity^9, 10^, difficulties of establishing multiplexed testing^11, 12^, and result interpretation through colorimetric/visual inspection which might be affected by technicians’ subjectivity^13^. Notably, SARS-CoV-2 LAMP tests have shown inadequate detection performances when testing low positive samples which exhibited Ct values over 30 on real-time PCR assays^14–16^; consequently, LAMP has been considered that having similar diagnostic accuracy as real-time PCR in detection of SARS-CoV-2 in the acute symptomatic phase of COVID-19 while the sample contains high viral load but not in early or late/clearance stages of the infection^17, 18^.

To solve those challenges, we developed a Peptide Nucleic Acid based Real Time-LAMP (PNA RT-LAMP) assay “AQ-TOP COVID-19 rapid Detection Kit Plus” which used dual labeled PNA probe that has been reported having superior specificity^19^ and sensitivity^20^ comparing to the accumulative dye such as SYBR green or sequence specific DNA fluorescent probes which are routinely used in LAMP and other molecular assays.

PNA is an artificial analog of DNA which the negatively charged phosphodiester backbone is replaced by a neutrally charged N-(2-aminoehtyl)-glycine backbone. The neutrally charged backbone makes PNA probe to bind its complementary nucleic acid with much higher affinity than the DNA detection probes, consequently, the sensitivity of PNA probe against the target nucleic acid is reported to be higher than the DNA probe^21, 22^. Furthermore, the specificity of the PNA is enhanced due to absence of the inter strand repulsion between PNA and its target nucleic acid^23^. Those particular biochemical properties make PNA suitable for use in many biological applications, especially as detection probes for PCR, FISH, multipurpose microarrays, and biosensors^24–27^.

In this study, the analytical and clinical performances of the PNA RT-LAMP assay against commercially available real-time PCR and colorimetric LAMP assays targeting SARS-CoV-2 were evaluated on both benchtop and portable real-time molecular amplifiers.

## Results

### Establishment of the PNA RT-LAMP assay

Two (2) sets of primer and PNA probes targeting two (2) specific regions in ORF1ab and N gene of SARS-CoV-2 were designed for amplification and detection of SARS-CoV-2. The sequences of the oligonucleotides were aligned against publicly available 56,303 SARS-CoV-2 sequences downloaded from NCBI database by February 2021 which contain full genomic information on CLC Main Workbench. All alignments showed 100% of identity against the queries showing that in silico analysis predicted that the assay can amplify and detect all SARS-CoV-2 isolates analyzed in this study. Results are summarized in Table 1, primer and probe sequences are shown in Supplementary Table S1.

**Table 1:** Summary of oligonucleotide sequence analysis.

Target	Oligonucleotide	Query with mismatch against 56,303 SARS-CoV-2 gRNA	Predicted inclusivity
ORF1ab	Primers (6 each)	0	100%
	Probe (1 each)	0	
N gene	Primers (6 each)	0	100%
	Probe (1 each)	0	

Sequences of all primer and probes showed no mismatch against the target queries (SARS-CoV-2 whole genomic sequences).

The assay targets one (1) specific region in each ORF1ab and N gene of the SARS-CoV-2 in two separate tubes with FAM fluorescence channel. Each tube contains primer and probe targeting human RNase P gene in HEX fluorescence channel as an internal control for parallel evaluation of sample quality/quantity and the test performance.

Both reverse transcription and LAMP reactions take place at 60 °C using the M-MLV Reverse Transcriptase and Bst DNA polymerase. During the amplification, dual labeled PNA probes can be incorporated into the amplification products. Upon the incorporation, fluorescence is generated and can be monitored by the fluorescence reader on the real-time PCR detection platforms in a real time fashion.

### The analytical specificity of the assay

The analytical specificity of the test was evaluated using 35 microorganisms shown in Table 2 which are frequently found in the human respiratory tract spiked in clinical negative nasopharyngeal (NP) swab specimen at concentrations of 10^6^ CFU/mL or higher for bacteria and 10^5^ pfu/mL or higher for viruses. In addition, RNA isolate from the SARS-CoV-2 negative human nasal wash was tested for specificity against the human normal nasal microflora. No detectable amplification curve was observed in FAM detection channel for SARS-CoV-2 ORF1ab and N genes, whereas the internal control RNase P in HEX detection channel did show 100% detection rate as expected in all three (3) test replicates for all organisms as well as for the nasal wash. Those results showed that the exclusivity of the assay against the microorganisms tested in this study is 100% (0% of false positivity) for both ORF1ab and N gene amplicon sets. Results are summarized in Table 2.

**Table 2:** Summary of cross reactivity study.

Microorganisms		Hit rate (#detected/#tested)				Result
		A well		B well		
		ORF1ab	RNase P	N	RNase P	
1	Human coronavirus 229E	0% (0/3)	100% (3/3)	0% (0/3)	100% (3/3)	Negative
2	Human coronavirus OC43	0% (0/3)	100% (3/3)	0% (0/3)	100% (3/3)	Negative
3	Human coronavirus HKU1	0% (0/3)	100% (3/3)	0% (0/3)	100% (3/3)	Negative
4	Human coronavirus NL63	0% (0/3)	100% (3/3)	0% (0/3)	100% (3/3)	Negative
5	SARS-coronavirus	0% (0/3)	100% (3/3)	0% (0/3)	100% (3/3)	Negative
6	MERS-coronavirus	0% (0/3)	100% (3/3)	0% (0/3)	100% (3/3)	Negative
7	Adenovirus type 1	0% (0/3)	100% (3/3)	0% (0/3)	100% (3/3)	Negative
8	Adenovirus type 2	0% (0/3)	100% (3/3)	0% (0/3)	100% (3/3)	Negative
9	Adenovirus type 3	0% (0/3)	100% (3/3)	0% (0/3)	100% (3/3)	Negative
10	Human Metapneumovirus	0% (0/3)	100% (3/3)	0% (0/3)	100% (3/3)	Negative
11	Parainfluenza virus 1	0% (0/3)	100% (3/3)	0% (0/3)	100% (3/3)	Negative
12	Parainfluenza virus 2	0% (0/3)	100% (3/3)	0% (0/3)	100% (3/3)	Negative
13	Parainfluenza virus 3	0% (0/3)	100% (3/3)	0% (0/3)	100% (3/3)	Negative
14	Parainfluenza virus 4	0% (0/3)	100% (3/3)	0% (0/3)	100% (3/3)	Negative
15	Influenza A (H3N2)	0% (0/3)	100% (3/3)	0% (0/3)	100% (3/3)	Negative
16	Influenza A (H1N1)	0% (0/3)	100% (3/3)	0% (0/3)	100% (3/3)	Negative
17	Influenza B	0% (0/3)	100% (3/3)	0% (0/3)	100% (3/3)	Negative
18	Enterovirus	0% (0/3)	100% (3/3)	0% (0/3)	100% (3/3)	Negative
19	Respiratory syncytial virus	0% (0/3)	100% (3/3)	0% (0/3)	100% (3/3)	Negative
20	Rhinovirus 1	0% (0/3)	100% (3/3)	0% (0/3)	100% (3/3)	Negative
21	Rhinovirus 14	0% (0/3)	100% (3/3)	0% (0/3)	100% (3/3)	Negative
22	Rhinovirus 7	0% (0/3)	100% (3/3)	0% (0/3)	100% (3/3)	Negative
23	*Chlamydia pneumoniae*	0% (0/3)	100% (3/3)	0% (0/3)	100% (3/3)	Negative
24	*Haemophilus influenzae*	0% (0/3)	100% (3/3)	0% (0/3)	100% (3/3)	Negative
25	*Legionella pneumophila*	0% (0/3)	100% (3/3)	0% (0/3)	100% (3/3)	Negative
26	*Mycobacterium tuberculosis*	0% (0/3)	100% (3/3)	0% (0/3)	100% (3/3)	Negative
27	*Streptococcus pneumoniae*	0% (0/3)	100% (3/3)	0% (0/3)	100% (3/3)	Negative
28	*Streptococcus pyogenes*	0% (0/3)	100% (3/3)	0% (0/3)	100% (3/3)	Negative
29	*Bordetella pertussis*	0% (0/3)	100% (3/3)	0% (0/3)	100% (3/3)	Negative
30	*Mycoplasma pneumoniae*	0% (0/3)	100% (3/3)	0% (0/3)	100% (3/3)	Negative
31	*Candida albicans*	0% (0/3)	100% (3/3)	0% (0/3)	100% (3/3)	Negative
32	*Pseudomonas aeruginosa*	0% (0/3)	100% (3/3)	0% (0/3)	100% (3/3)	Negative
33	*Staphylococcus epidermis*	0% (0/3)	100% (3/3)	0% (0/3)	100% (3/3)	Negative
34	*Streptococcus salivarius*	0% (0/3)	100% (3/3)	0% (0/3)	100% (3/3)	Negative
35	*Staphylococcus aureus*	0% (0/3)	100% (3/3)	0% (0/3)	100% (3/3)	Negative
36	*Human nasal wash*	0% (0/3)	100% (3/3)	0% (0/3)	100% (3/3)	Negative

RNA extracts from all tested microorganisms as well as direct human nasal wash were confirmed to not cross-react with PNA RT-LAMP assay.

### Clinical performance of the assay against commercial Real-time PCR test

A clinical evaluation of the PNA RT-LAMP assay was performed that evaluating a total of 270 blinded clinical NP swab specimens including 70 SARS-CoV-2 positive and 200 negative individual, leftover, de-identified specimens collected in the Chungnam National University Hospital which were previously tested using commercially available FDA EUA authorized real-time PCR test targeting SARS-CoV-2 specific RdRp and E genes (PowerCheck 2019-nCoV real-time PCR kit, Kogene Biotech). Positive samples were divided into 2 groups based on the Ct values exhibited on the real-time PCR: 1) High positives: a total of 44 samples which both targets showed Ct ≤ 30; 2) Low positives: a total of 26 samples which at least one of the targets showed Ct > 30. Both clinical sensitivity and specificity of the PNA RT-LAMP assay against the real-time PCR test result were confirmed to be 100% for both positive groups (Sensitivity 95% CI: 94.80%-100.00%; Specificity 95% CI: 98.10%-100.00%). Mean Ct values of the high and low positive groups on the real-time PCR were 22.95 and 31.53 for RdRp; 22.62 and 31.42 for N gene, while mean Tt (Threshold time) on the RT LAMP were 10.18 and 18.28 for ORF1ab; 9.39 and 15.56 for N gene, respectively. This result showed that the clinical performances of the 2 assays are identical, whereas the analysis time of RT-LAMP is quite shorter than the real-time PCR. The results of the clinical evaluation are summarized in Table 3.

**Table 3:** Summary of clinical evaluation results.

(a) PNA RT-LAMP test AQ-TOP COVID-19 rapid detection kit plus showed 94.87–100.00% (95% CI) of clinical sensitivity and 98.17–100.00% (95% CI) of clinical sensitivity when testing against the real-time PCR test				
	Comparator assay (Real-time PCR test)			
	Positive	Negative	Total	
**AQ-TOP™ COVID-19 Rapid Detection Kit Plus**				
Positive	70	0	70	
Negative	0	200	200	
Total	70	200	270	
Clinical sensitivity	100% (70/70); 95% CI 94.87–100.00%			
Clinical specificity	100% (200/200); 95% CI 98.17–100.00%			

**(b) Ct and Tt comparison of clinical positive samples**				
	Mean Ct (real-time PCR)		Mean Tt (RT LAMP)	
	ORF1ab	N gene	RdRp	N gene
High positives	22.95	22.62	10.18	9.39
Low positives	31.53	31.42	18.28	15.56

### Comparative sensitivity of the PNA RT-LAMP assay and other EUA authorized SARS-CoV-2 molecular tests

The analytical sensitivity of the PNA RT-LAMP assay was evaluated using RNA extracts from heat-inactivated SARS-CoV-2 (USA-WA1/2020, ZeptoMetrix, USA) at tenfold dilution series spiked in clinical negative NP swabs comparing with commercially available FDA EUA authorized real-time RT-PCR test (SS-9930, Seasun Biomaterials) and Colorimetric LAMP SARS-CoV-2 assay (E2019S, NEB). All three methods showed identical analytical sensitivities which exhibited the lowest detection limit of approximately 1 genomic copy of SARS-CoV-2 per microliter of RNA extract, indicating that the analytical sensitivity of the PNA RT-LAMP assay was comparable to the real-time PCR and traditional colorimetric LAMP methods (Table 4, Supplementary Fig. S1).

**Table 4:** Comparative analysis of RT-LAMP, real-time PCR and colorimetric LAMP assays.

RNA concentration	Real-time RT-PCR					PNA RT-LAMP					Colorimetric LAMP		
	ORF1ab		N gene		Result	ORF1ab		N gene		Result	N/E genes		Result
	Hit rate	Mean Ct	Hit rate	Mean Ct		Hit rate	Mean Tt	Hit rate	Mean Tt		Hit rate	Color	
10,000 cp/µL	5/5	21.14	5/5	20.12	Positive	5/5	7.10	5/5	6.96	Positive	5/5	Yellow	Positive
1,000 cp/µL	5/5	24.68	5/5	25.25	Positive	5/5	8.42	5/5	7.57	Positive	5/5	Yellow	Positive
100 cp/µL	5/5	28.99	5/5	28.54	Positive	5/5	10.66	5/5	9.65	Positive	5/5	Yellow	Positive
10 cp/µL	5/5	32.30	5/5	32.65	Positive	5/5	11.39	5/5	11.37	Positive	5/5	Yellow	Positive
1 cp/µL	5/5	36.65	5/5	36.11	Positive	5/5	12.19	5/5	12.80	Positive	5/5	Yellow	Positive
0.1 cp/µL	0/5	ND	0/5	ND	Negative	0/5	ND	0/5	ND	Negative	0/5	Red	Negative

All three methods exhibited the lowest detection limits capable of detecting SARS-CoV-2 genomic RNA of 1 genomic copy (cp) per µL of RNA extract. All three assays could not detect dilution series contain 0.1 cp/µL of SARS-CoV-2 RNA.

For further evaluation, positive detection rates of PNA RT-LAMP and Colorimetric LAMP assays were evaluated using 15 clinical individual positive NP swabs including five (5) high positives which exhibited Ct values up to 30 cycles, five (5) moderate positives which exhibited Ct values between 31 to 34 cycles, and five (5) low positives which exhibited Ct values higher than 35 cycles for both SARS-CoV-2 ORF1ab and N genes that were previously identified using the Real-time RT PCR test (SS-9930, Seasun Biomaterials). The PNA RT-LAMP assay successfully detected all 15 samples from the three positive groups whereas the colorimetric LAMP test has missed 2 low positives which exhibited Ct values over 37 cycles for both ORF1ab and N genes on the real-time PCR assay (Table 5, Supplementary Fig. S2).

**Table 5:** Comparative table of clinical positive samples testing.

Sample		Real-time RT-PCR					PNA RT-LAMP										Colorimetric LAMP		
							Testing on CFX-96					Testing on SMARTAMP							
		ORF1ab		N gene		Result	ORF1ab		N gene		Result	ORF1ab		N gene		Result	N/E genes		
		Ct	Hit rate	Ct	Hit rate		Tt	Hit rate	Tt	Hit rate		Tt	Hit rate	Tt	Hit rate		Color	Hit rate	Result
High positives	1	23.5	5/5 (100%)	22.7	5/5 (100%)	Positive	9.0	5/5 (100%)	7.5	5/5 (100%)	Positive	8.9	5/5 (100%)	8.1	5/5 (100%)	Positive	Yellow	5/5 (100%)	Positive
	2	26.9		25.7		Positive	9.0		7.5		Positive	9.2		8.0		Positive	Yellow		Positive
	3	20.5		19.2		Positive	8.5		6.8		Positive	8.4		7.2		Positive	Yellow		Positive
	4	21.4		20.3		Positive	8.1		6.6		Positive	8.6		6.5		Positive	Yellow		Positive
	5	27.3		26.3		Positive	9.6		8.1		Positive	9.2		7.5		Positive	Yellow		Positive
Moderate positives	1	33.6	5/5 (100%)	31.71	5/5 (100%)	Positive	14.2	5/5 (100%)	11.1	5/5 (100%)	Positive	13.8	5/5 (100%)	10.5	5/5 (100%)	Positive	Yellow	5/5 (100%)	Positive
	2	34.5		33.3		Positive	14.3		11.0		Positive	14.5		12.3		Positive	Yellow		Positive
	3	33.9		32.0		Positive	13.8		10.9		Positive	14.0		11.6		Positive	Yellow		Positive
	4	33.4		31.5		Positive	13.2		9.9		Positive	13.5		10.4		Positive	Yellow		Positive
	5	33.5		32.8		Positive	13.0		11.1		Positive	13.8		9.5		Positive	Yellow		Positive
Low positives	**1**	37.1	5/5 (100%)	37.1	5/5 (100%)	Positive	24.0	5/5 (100%)	12.3	5/5 (100%)	Positive	22.5	5/5 (100%)	14.1	5/5 (100%)	Positive	**Red**	3/5 (60%)	**Negative**
	2	37.1		35.7		Positive	15.6		12.9		Positive	17.2		13.6		Positive	Yellow		Positive
	3	36.5		35.8		Positive	22.1		11.7		Positive	20.1		14.3		Positive	Yellow		Positive
	4	36.1		35.5		Positive	14.8		15.0		Positive	16.3		11.5		Positive	Yellow		Positive
	**5**	37.2		36.6		Positive	22.6		13.7		Positive	23.4		12.5		Positive	**Red**		**Negative**

15 samples of high, moderate, and low positive groups exhibited positive amplification signals when tested using the PNA RT-LAMP assay on both CFX-96 real-time PCR detection system and a portable isothermal amplifier SMARTAMP. Colors of all 10 samples of high and moderate positive groups turned into yellow or orange indicating the amplification of SARS-CoV-2 targets, while two (2) low positive NP swabs (sample #1, 5 bold in the table) out of the five (5) could not be detected as positive.

Those results show that the sensitivity of the PNA RT-LAMP assay is higher than the colorimetric LAMP assay and identical to the real-time PCR method even testing the low positive samples showed late amplification rates on the real-time PCR method.

### PNA RT-LAMP testing on a portable isothermal amplifier

**Figure 1:**
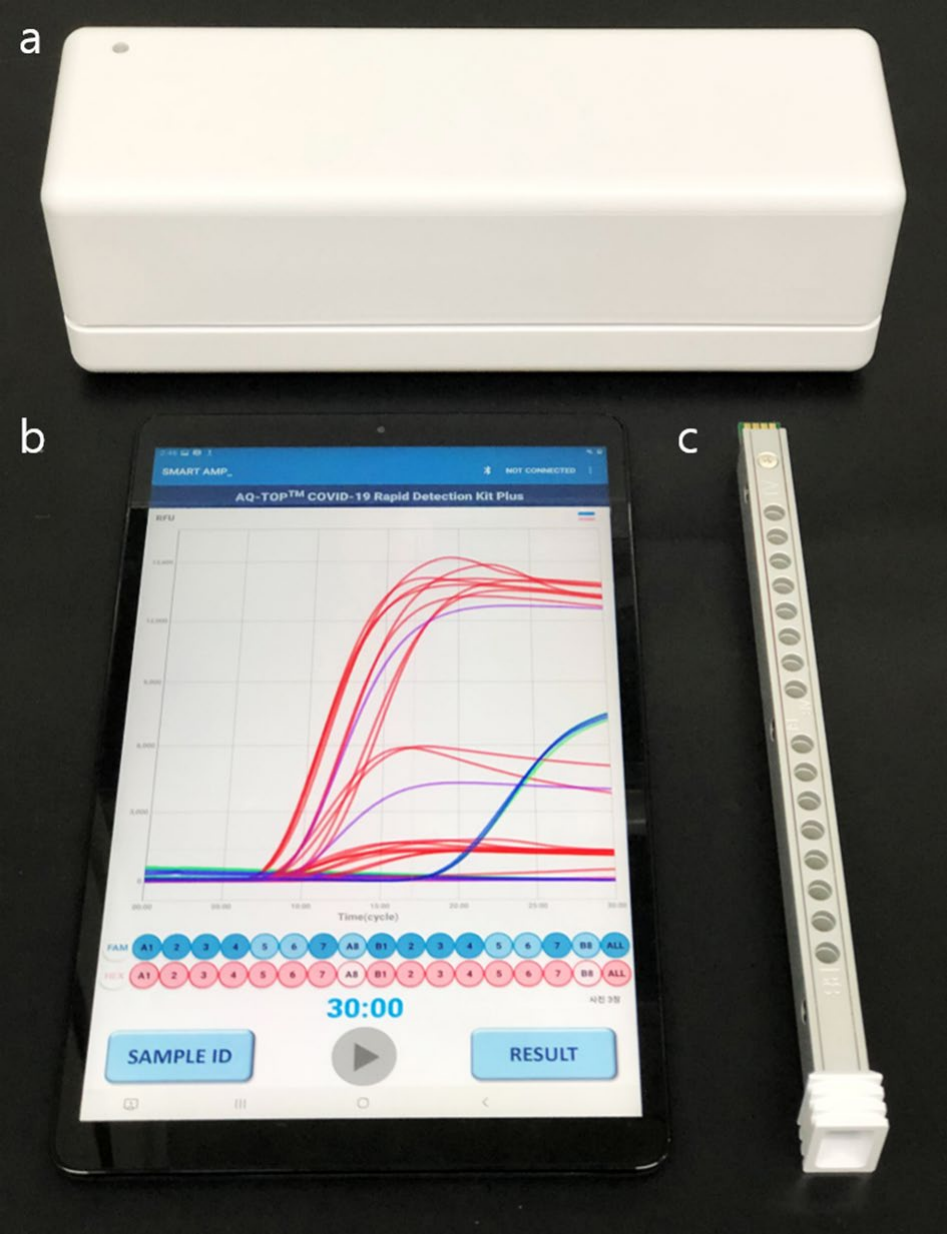
(**a**) A portable isothermal amplifier, (**b**) tablet computer with an operating system and c) 16 well heating plate applicable to the amplifier.

Finally, we tested the 15 clinical high, moderate, low positive NP swabs on the PNA RT-LAMP test using a portable isothermal amplifier SMARTAMP (SS-7010, Seasun Biomaterials) which could collect fluorescence signals of FAM and HEX (excitation at 490–540 and emission at 515–555) reporter dyes in a real-time manner. The device is fully portable and compatible with tablet computers with an easy-to-use operating system that can be applicable at POC testing (Fig. 1). All 15 samples with various viral loads were detectable 100% within 15 min when testing with the same run condition as in the real-time PCR instrument (Table 5). This data shows that the PNA RT-LAMP assay can be applicable at POC testing even further evaluations with increasing clinical sample numbers are required.

## Discussion

Here, we developed and evaluated analytical, clinical performances of PNA based RT-LAMP assay targeting ORF1ab and N genes of SARS-CoV-2. However, the LAMP has been known as having high and rapid amplification efficiency which makes its analytical sensitivity comparable to real-time PCR, high rate of false positivity while testing field clinical samples has been reported on the strength of the result interpretation method based on pH dependent colorimetric visualization^28, 29^. Since the colorimetric display of traditional LAMP is based on the principle of color change reaction of pH indicators such as phenol red^30^, the result is significantly affected by remnants from nucleic acid extraction reagents contained in the clinical sample elutes as well as the LAMP reaction buffers and enzyme contents^31^. To overcome those issues, we applied dual-labeled PNA as a detection probe in LAMP reaction for fluorescence detection of the amplification product in a real-time manner. PNA has been reported that having superior specificity against its template nucleic acid with its neutrally charged peptide backbone nature which does not have a nonspecific binding affinity with minus charged natural phosphate backbone of the template nucleic acid^22, 32^. The un-cleavable peptide backbone also reduces the risk of non-specific signal production as a result of thermal degradation during long-term incubation at elevated temperatures.

We confirmed that the PNA RP-LAMP assay does not cross-react with non-target microorganisms at high concentrations. Also, both clinical specificity and sensitivity against real-time RT PCR assay showed 100% of accuracies. The above results showed that PNA RT-LAMP assay employs high enough analytical specificity as well as the clinical performances are comparable to the gold standard real-time PCR method.

Results of the sensitivity testing using tenfold dilution series of SARS-CoV-2 inactivated isolate demonstrated the potential of the PNA RT-LAMP test could detect ~ 1 copy of template RNA per microliter of the sample within approximately 12 min, however, the next tenfold dilution series contain approximately 0.1 copy of the SARS-CoV-2 genomic material could not be detected even in reaction up to 30 min. Practically, every tenfold dilution series in real-time PCR exhibit the Ct values of 3.3 apart while PNA RT-LAMP (in this study) exhibited one (1) minute lateness for every tenfold dilution series. According to these practices, the dilution series contain ~ 0.1 genomic copies could be detected on PNA RT-LAMP assay although it couldn’t. Our assumption on this was the most amount of Bst polymerase has already been consumed during amplification of an internal control RNase P, and not enough concentration of active enzymes remained for amplification of the SARS-CoV-2 in extremely low concentration. Because RNase P showed amplification signals at around 15–20 Tt on the RT LAMP while the SARS-CoV-2 targets near to the test LOD showed Tt around 12–13 (Supplementary Fig. S1). We observed that once RNase P amplification has started SARS-CoV-2 targets were not amplified. To test this assumption, we did further tests by increasing the Bst concentration up to tenfold, reducing the primer/probe concentrations of the internal control, however the expected result has not been obtained.

The final goal of this study was the development of a rapid molecular detection method that can be applicable at POC testing while having a comparable clinical performance with the gold standard real-time PCR method. We have confirmed that the PNA RT-LAMP assay can detect low positive samples contain a few copies of target RNA that exhibiting Ct values > 35 on the real-time PCR although traditional LAMP has missed those samples. This result was reproducible on a both benchtop real-time PCR detection system and a portable isothermal amplification device.

However, the PNA RT-LAMP assay developed in this study is currently applicable with RNA extracts from clinical NP swabs, we have been working on optimization of the assay on use of saliva and direct NP swabs without an additional sample preparation step which is more suitable at POC testing and in low-resource settings.

## Methods

### Materials used in this study

2 × LAMP Master Mix contains M-MLV Reverse Transcriptase and Bst DNA polymerase were purchased from Elpis-Biotech, Korea. Oligonucleotides and dual labeled PNA probes were synthesized in Cosmo Genetech, Korea, and Panagene, Korea respectively. Heat inactivated SARS-CoV-2 isolate USA-WA1/2020 was purchased from ZeptoMetrix, USA. Strains of the microorganisms used in the specificity testing were purchased from Korean Bank of Pathogenic Viruses and the National Culture Collection for Pathogens, Korea.

### Sequence analysis

A total of 12 primer and 2 PNA probe sequences were analyzed against 56,303 SARS-CoV-2 genomic sequences contain whole genome information downloaded from GenBank (https://www.ncbi.nlm.nih.gov/) on CLC Main Workbench 9.5.2 with molecular biology tool “Find binding sites and create fragment”.

### Sample preparation

For analytical sensitivity study, RNAs were purified from 300 µL of NP swabs spiked in tenfold dilutions series of SARS-CoV-2 inactivated isolate (ZeptoMetrix, USA-WA1/2020) using TOP Viral DNA/RNA extraction kit (Seasun Biomaterials, SS-1300) according to the manufacturer’s instructions and eluted in 30 µL of elution buffer included in the kit. Genomic copies per µL were previously quantified using NanoDrop values of the nucleic acid extract of undiluted SARS-CoV-2 isolate as a formula in below:

$$Genomic\, copies=\frac{ng \,of \,single \,stranded \,RNA \times Avagadr{o}'s\,constant \,(6.02\times {10}^{23})}{Length \,of \,nucleotide \times {10}^{9} \times 325 \,Daltons}$$

The size of the SARS-CoV-2 reference genome (NCBI Reference Sequence: NC_045512.2) is assumed to be 29,903 bp ss-RNA was used in the calculations. For comparative clinical sensitivity and clinical evaluation, 300 µL clinical positive NP swab specimens were processed using PANAMAX48 viral DNA/RNA extraction kit (Panagene, PNAK 1001) on PANAMAX nucleic acid automated extractor following the manufacturer’s instructions. Each RNA isolate was used immediately after the extraction.

### PNA RT-LAMP amplification and detection

PNA RT-LAMP test was performed with a total of 30 µL of total reaction volume using 15 µL of reaction buffer, 1 µL of enzyme mix, 4 µL of the reaction mix, and 10 µL of template RNA on CFX-96 real-time PCR detection system or SMARTAMP portable isothermal amplifier with the run condition of 60 degrees Celsius for 30 min with fluorescence signal collection at every 1 min. Samples that exhibited positive amplification signals within 30 min on FAM fluorescence channel in at least one reaction well was defined as positives. Samples that did not produce positive FAM signal while HEX detection channel regarding endogenous quality control RNase P produced amplification curve were defined as negatives.

### Clinical evaluation

RNAs were extracted from 200 µL of individual, leftover, de-identified nasopharyngeal swab specimens collected in the Chungnam National University Hospital previously tested with FDA authorized under EUA SARS-CoV-2 real-time PCR assay PowerCheck 2019-nCoV real-time PCR kit (Kogene Biotech, Korea) targeting SARS-CoV-2 specific RdRp and E genes. 10 µL of RNA extracts were tested for each reaction mixtures of the PNA RT-LAMP assay in a blinded manner on the CFX-96 real-time PCR detection system. Clinical accuracies were calculated by using the standard method in the base of CI 95%^33^.

### Sensitivity testing

RNA extracts were tested with five (5) individual extraction replicates on the PNA RT-LAMP assay, SARS-CoV-2 real-time RT-PCR assay U-TOP COVID-19 Detection Kit (SS-9930, Seasun Biomaterials) targets ORF1an/RdRp and N gene of SARS-CoV-2 with human RNase P in a single tube; and SARS-CoV-2 Rapid Colorimetric LAMP Assay Kit (E2019S, NEB) targets SARS-CoV-2 E/N genes in one tube, and rActin in a separate tube for the quality control according to the instructions provided from the manufacturers. Results were interpreted according to the instructions supplied from the manufacturers as briefly the samples which exhibited Ct values less than 38 for at least one SARS-CoV-2 target gene were defined as positives for the real-time PCR assay, and samples which color of the reaction mixture turned into yellow or light orange from red after completion of the LAMP reaction were defined as positives for the colorimetric LAMP assay. Real-time PCR was performed on the CFX-96 real-time PCR detection system, SARS-CoV-2 Rapid Colorimetric LAMP Assay was performed using ABI2720 thermal cycler (Applied Biosystems, USA). PNA RT-LAMP test was performed on both CFX-96 real-time PCR detection system and SMARTAMP isothermal amplifier with the same experimental condition.

### Source of clinical specimens and ethnics statement

Clinical samples collected in Chungnam National University Hospital at the first wave of COVID-19 (February to April 2020) in the Republic of Korea were used in this study. Handling and analysis of the clinical samples were approved by the Institutional Review Board (IRB. CNUH2020-06-123) of Chungnam National University Hospital. Informed consent was obtained from all participants and the research was performed on anonymized, de-identified RNA samples following the Declaration of Helsinki.

## Supplementary Information

Supplementary Information.
