# Supplementary material for: Development, evaluation of the PNA RT-LAMP assay for rapid molecular detection of SARS-CoV-2
Source: Sci Rep. 2021 Oct 14;11:20471. doi: 10.1038/s41598-021-00041-y (PMC8516927; doi:10.1038/s41598-021-00041-y)

**Supplementary table 1.** Sequences of the primers and probes used in this study

| Target | | Name | Sequence (5’-3’) |
| --- | --- | --- | --- |
| Primer | ORF1ab | AB_F3 | GAATTATGGCCTCACTTG |
|  |  | AB_B3 | ACCATCAGTAGATAAAAG |
|  |  | AB_FIP | TCACTCAATACTTGAGCACACTCACATACAACGTGTTGTAGC |
|  |  | AB_BIP | GTCATGTGTGGCGGTTCACTAAACACTATTAGCATAAGCAGTTG |
|  |  | AB_LPF | CTATAGAAACGGTGTGACA |
|  |  | AB_LPB | GTTAAACCAGGTGGAAC |
|  | N gene | N_F3 | CAAGAAATTCAACTCCAGGC |
|  |  | N_B3 | AGTACGTTTTTGCCGAGG |
|  |  | N_FIP | GCAAAGCAAGAGCAGCATCAAGTAGGGGAACTTCCCTG |
|  |  | N_BIP | ACAGATTGAACCAGCTTGAGAGGCAGCAGATTTCTTAGTGAC |
|  |  | N_LPF | CATTGCCAGCCATTCTAG |
|  |  | N_LPB | CCAACAACAACAAGGCCA |
|  | RNase P | RP_F3 | CTACATTCACGGCTTGGGC |
|  |  | RP_B3 | GGGGATAAGTGGAGGAGTGT |
|  |  | RP_FIP | TTGGCAGCCACCTGCAAGGAATCAACCGCGCCATCAAC |
|  |  | RP_BIP | CGACACACGGGAGCCACTGATGACCCTGAAGACTCGGATG |
|  |  | RP_LPF | CAGCTGCAGCGCGATG |
|  |  | RP_LPB | GACTCGGATCCGCAACA |
| Probe | ORF1ab | AB_P | Dabcyl – CATCAGGAGATGCCA – FAM |
|  | N gene | N_P | Dabcyl – CAAAATGTATGGTA – HEX |
|  | RNase P | R_P | Dabcyl – ACTCAGCCATCCAC – Texas red |

**Supplementary figure 1. Comparison of the analytical sensitivities of real-time RT-PCR, PNA RT-LAMP and Colorimetric LAMP assays.** A) Real-time PCR assay result. B) PNA RT-LAMP assay result. C) Analytical sensitivity of Colorimetric LAMP assay. All three methods showed positive amplifications when testing serially diluted samples contain approximately 10,000 to 1 cp/µL SARS-CoV-2 RNA template while samples contain ~ 0.1 cp/µL of template RNA were not able to be amplified.


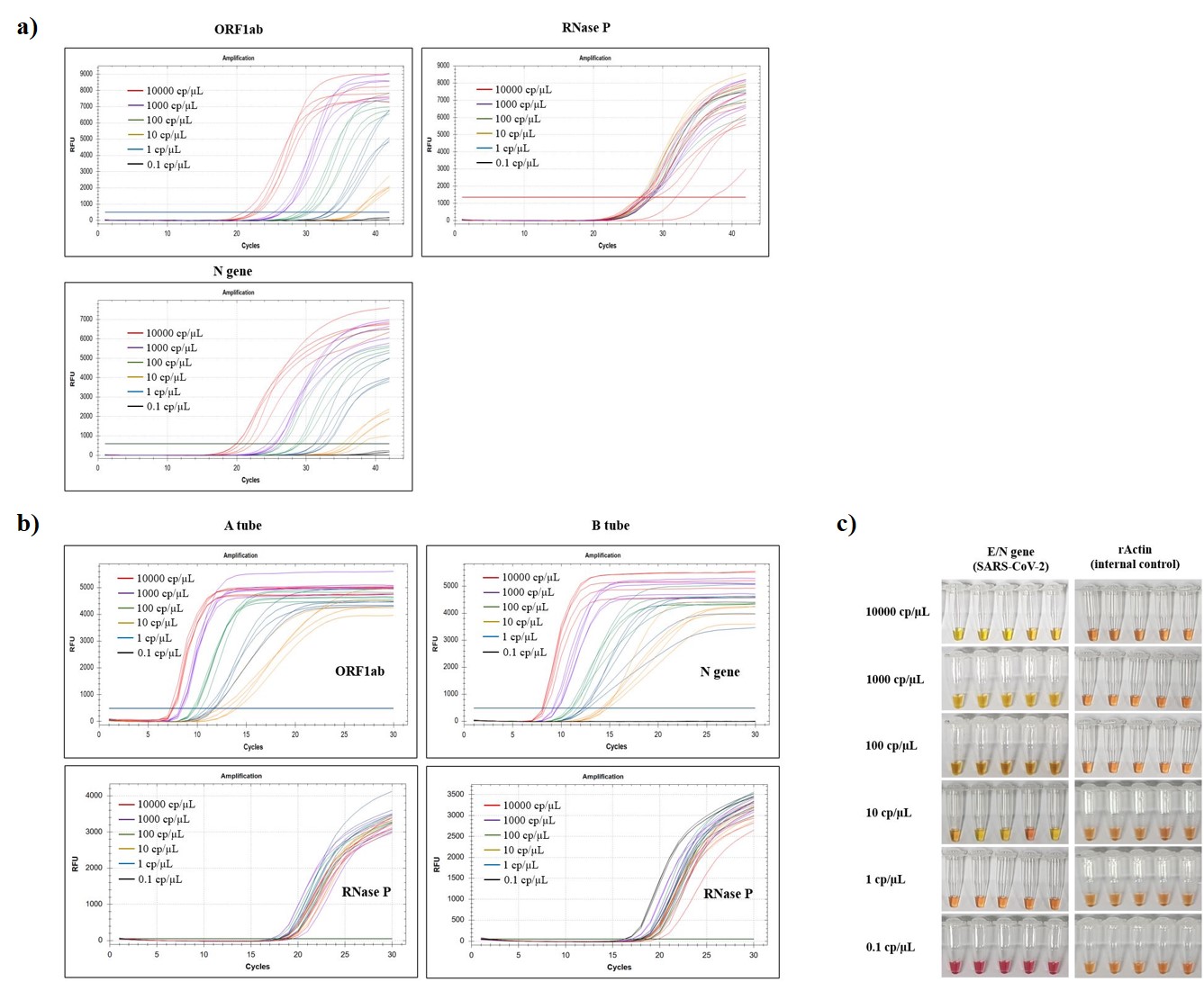


**Supplementary figure 2. Comparative analysis of the sensitivities of SARS-CoV-2 molecular assays using clinical positive NP swabs including high, moderate and low positives.** A) Results of the real-time PCR assay tested 15 samples of 3 positive groups. B) Results of the PNA RT-LAMP assay on CFX-96 real-time PCR detection system. All 15 samples of 3 positive groups exhibited amplification curves less than 30 for both ORF1ab and N gene targets. C) Results of PNA RT-LAMP assay on a portable isothermal amplifier SMARTAMP. All 15 samples exhibited amplification curves less than 30 for both ORF1ab and N gene targets. D) Results of the Colorimetric LAMP assay. Colors of all 10 samples of high and moderate positive groups turned into yellow or orange indicating the amplification of SARS-CoV-2 targets, while two (2) low positive NP swabs (sample #1, 5) out of the five (5) could not been detected as positive. An internal control rActin showed positive amplification signal for 15 samples.


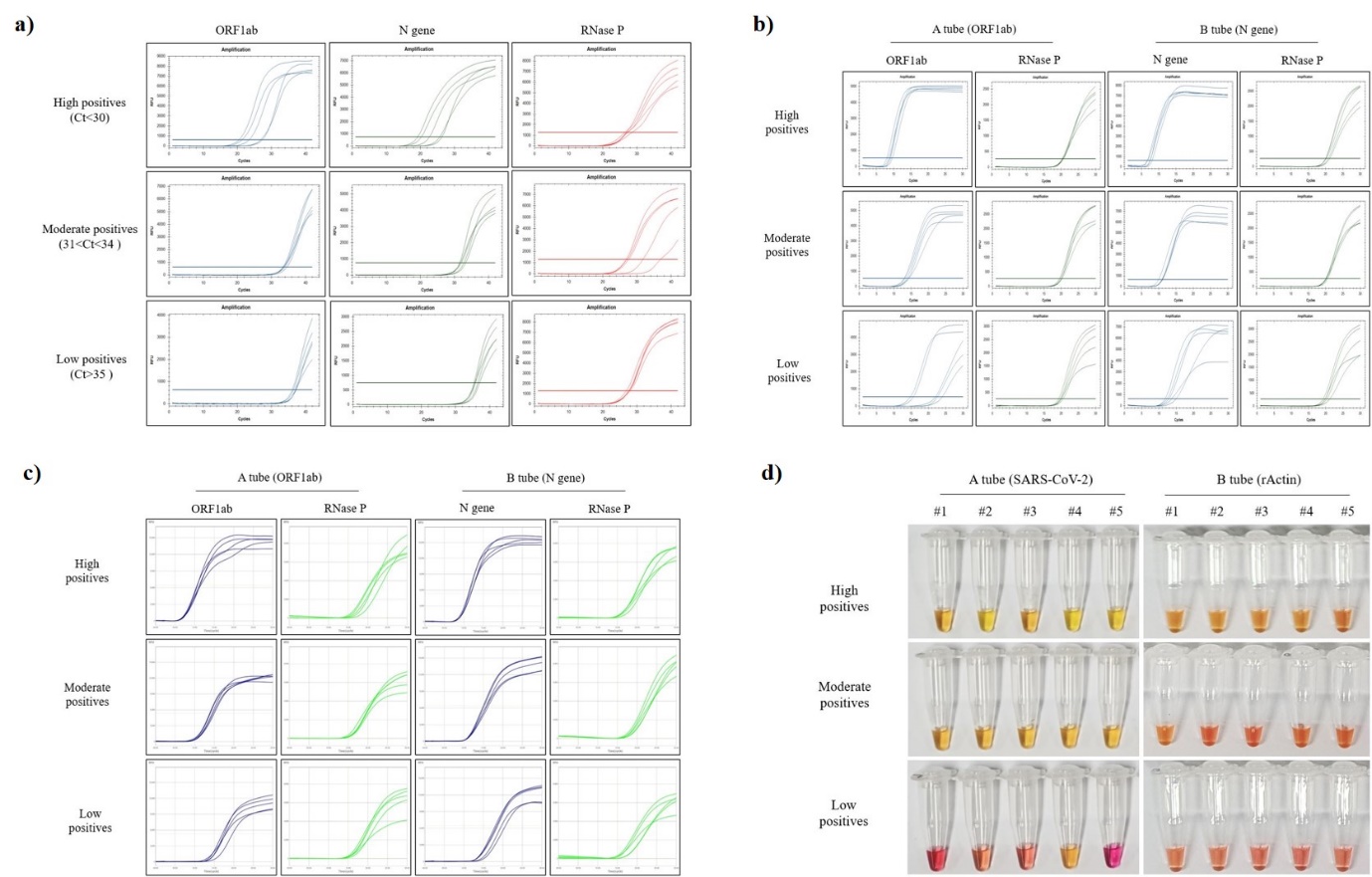

Supplement: Supplementary file 1 — Supplementary Information. [file 41598_2021_41_MOESM1_ESM.docx]
